# Acute Pericarditis Triggered by Severe Thyrotoxicosis

**DOI:** 10.7759/cureus.65941

**Published:** 2024-08-01

**Authors:** Khalid Javed, Shaikh B Iqbal, Usman Sagheer, Shiavax J Rao

**Affiliations:** 1 Internal Medicine, MedStar Union Memorial Hospital, Baltimore, USA

**Keywords:** pericardial effusion, echocardiogram, echocardiography, pericardial disease, thyroid disease, thyrotoxicosis, pericarditis

## Abstract

Acute pericarditis is a common inflammatory disorder with several causes including infection, malignancy, acute myocardial infarction, and autoimmune disease. Acute pericarditis can rarely present in the setting of thyrotoxicosis. A 65-year-old man with a past medical history of HIV, diastolic dysfunction, and prediabetes presented with positional chest pain, respiratory distress, and altered mentation. He was found down on the ground in a lethargic state and was last seen normally five days before the presentation. On presentation, he was tachycardic and tachypneic, requiring supplemental oxygenation with a nonrebreather mask to maintain adequate oxygen saturation. Initial electrocardiogram (EKG) demonstrated diffuse ST-elevations with early repolarization, consistent with acute pericarditis. Laboratory diagnostics revealed elevated lactic acid, leukocytosis, acute kidney injury, undetectable thyroid stimulating hormone, and elevations in T3, T4, C-reactive protein, brain natriuretic peptide, and creatinine kinase. Given the patient's complex presentation involving thyrotoxicosis and pericarditis, a multidisciplinary team discussion was pursued involving critical care, cardiology, and endocrinology. He was started on intravenous methylprednisolone (subsequently transitioned to prednisone), methimazole, and metoprolol. Colchicine was subsequently added for the management of pericarditis and prednisone was continued (given concomitant thyroid disease) with a plan for tapering them off, per cardiology and endocrinology recommendations. A transthoracic echocardiogram revealed a small pericardial effusion. Anticoagulation was not initiated given the potential risk of developing a hemorrhagic pericardial effusion. Thyroid ultrasound was nonsuggestive of Graves’ disease. Thyrotoxicosis may present with a constellation of symptoms, including acute pericarditis. Timely recognition with EKG and echocardiography can aid in prompt management.

## Introduction

Thyroid disease is well known to affect the cardiovascular system in numerous ways, including arrhythmias, hypertension, and heart failure. Pericardial involvement is often seen in hypothyroidism with an incidence rate of 3% to 37% [1]. Acute pericarditis is a common inflammatory disorder with several causes including infection, malignancy, acute myocardial infarction, and autoimmune diseases [2]. Acute pericarditis can rarely present in the setting of thyrotoxicosis based on limited literature in the form of case reports. The mechanism for pericardial involvement in Graves’ disease is not well understood. We present an interesting clinical case of acute pericarditis triggered by severe thyrotoxicosis.

## Case presentation

A 65-year-old man was found down in his apartment, covered in urine and feces, and last seen normally five days prior. He had a past medical history of heart failure with preserved ejection fraction, HIV (on antiretroviral therapy), hepatitis C virus infection, chronic obstructive pulmonary disease (COPD) requiring oxygen supplementation, obstructive sleep apnea requiring nocturnal continuous positive pressure ventilation, hypertension, chronic kidney disease, substance use disorder (in remission), and prediabetes. On initial evaluation by emergency medical services, he had labored breathing and constricted pupils. His mentation improved following administration of naloxone; however, he complained of dull, aching substernal chest pain exacerbated by deep inspiration. He stated he had a cyclical eating pattern associated with a significant weight loss of 20 pounds in three weeks and multiple days of diarrhea. He denied any dyspnea, palpitations, tremors, or changes in his hair or skin.

On presentation, he was hypertensive (142/75 mmHg), tachycardic (151 beats per minute), tachypneic (26 breaths per minute), and required supplemental oxygenation with a nonrebreather mask. He was in moderate respiratory distress requiring the use of accessory muscles and had diffuse inspiratory and expiratory wheezing, as well as an irregularly irregular cardiac rhythm. A skin exam demonstrated a linear abrasion with a contusion on the right lower thorax. He had no evidence of thyromegaly, thyroid tenderness or nodularity, tremors, or hyperreflexia. 

Initial laboratory diagnostics are summarized in Table 1. An infectious workup was also completed and was found to be negative. Previous HIV workup three months before admission demonstrated HIV RNA viral load less than 20 (less than 20-75 copies/mL) and within normal CD4 counts.

**Table 1:** Initial laboratory diagnostic test results. NT-proBNP, N-terminal pro-b-type natriuretic peptide; TSH, thyroid-stimulating hormone; T4, thyroxine; T3, triiodothyronine; CRP, c-reactive protein

Laboratory test	Result	Reference range
TSH	<0.005 uIU/mL	0.4-4.000 uIU/mL
Free T4	3.4 ng/dL	0.76-1.46 ng/dL
T3	5.6 ng/dL	2.18-3.98 ng/dL
Creatinine kinase	823 units/L	39-308 units/L
Lactic acid	3.6 mmol/L	0.7-2 mmol/L
Leukocyte count	21.6 k/uL	4-10.8 k/uL
CRP	135 mg/L	0-3 mg/L
NT-proBNP	2,500 pg/mL	<125 pg/mL
Troponin-I	<0.015 ng/mL	0.000-0.045 ng/mL

**Figure 1:**
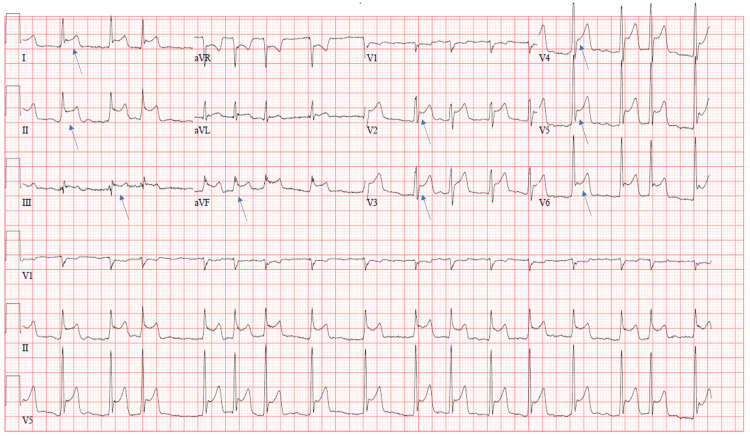
Initial 12-lead electrocardiogram revealing diffuse ST elevations (blue arrows).

His initial cardiac rhythm was atrial flutter with intermittent episodes of atrial fibrillation. A 12-lead electrocardiogram (EKG) revealed diffuse ST elevations (Figure 1). A plain film chest radiograph demonstrated hyperinflation, while CT angiography of the chest ruled out any acute abnormalities, including pulmonary embolism. An initial transthoracic echocardiogram revealed normal left ventricular cavity size, normal posterior wall thickness, and normal septal wall thickness. Left ventricular ejection fraction could not be assessed due to poor image quality, and diastolic function could not be assessed due to atrial fibrillation. He was admitted to the ICU for further management of his altered mental status, poor respiratory drive from opioid use, and thyrotoxicosis with resultant atrial fibrillation with rapid ventricular response.

Given the patient's complex presentation involving thyrotoxicosis and pericarditis, a multidisciplinary team discussion was pursued involving critical care, cardiology, and endocrinology. He was started on intravenous methylprednisolone (subsequently transitioned to prednisone), methimazole, and metoprolol. Colchicine was subsequently added for the management of pericarditis, and prednisone was continued (given concomitant thyroid disease) with a plan for tapering them off, per cardiology and endocrinology recommendations. Endocrinology subsequently recommended decreasing his home methimazole dose and switching beta blockade to propranolol, to prevent peripheral T4 conversion. Thyroid-specific antibodies were not remarkable. Thyroid ultrasound did not reveal any characteristic findings for Graves’ disease. Despite his high CHA_2_DS_2_-VASc score, cardiology recommended against anticoagulation, given the high risk of pericarditis-related hemorrhagic effusion.

**Figure 2:**
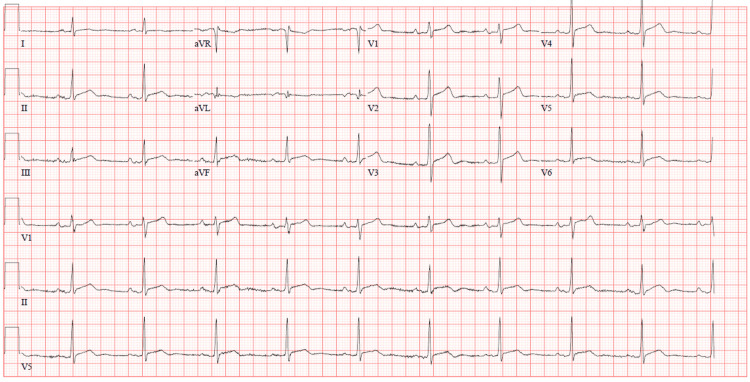
Interval EKG showing normal sinus rhythm with resolution of previously noted diffuse ST elevations. EKG, electrocardiogram

**Figure 3:**
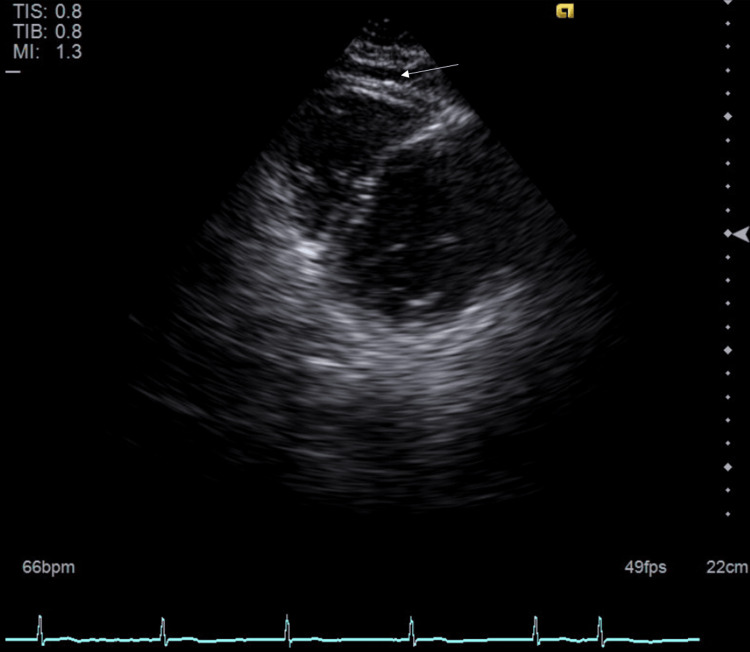
Transthoracic echocardiogram revealing preserved ejection fraction of 55%-60% and a small pericardial effusion (white arrow).

His symptoms eventually improved, and subsequent EKGs demonstrated that his rhythm had converted to normal sinus rhythm with improvement in the previously noted ST changes (Figure 2). Free thyroxine (T4) levels continued to decrease after adjustments in his methimazole dose. Propranolol was switched to metoprolol once his free T4 levels were within the normal range. A repeat transthoracic echocardiogram demonstrated a preserved ejection fraction of 55% to 60%, normal estimated right atrial pressure, and a small pericardial effusion without tamponade characteristics (Figure 3). 

The patient was discharged to a subacute rehabilitation facility. He continued on a lower dose of methimazole, colchicine, and metoprolol. He was recommended to follow up with endocrinology as an outpatient for his thyroid disease.

## Discussion

Acute pericarditis refers to inflammation of the pericardium. It may be a manifestation of an isolated process or an underlying systemic disease. It is found in about 0.2% of hospitalized patients admitted for non-ischemic chest pain and occurs more commonly in men [3,4]. The more common cardiac complications are atrial fibrillation, congestive heart failure, mitral valve dysfunction, and atrioventricular block [5]. Pericardial effusion and acute pericarditis are exceedingly rare complications of thyrotoxicosis. The mechanism is hypothesized to be due to either autoimmunity or viral illness leading to antibody production causing autoinflammation [6].

Patients typically present with sudden, sharp, pleuritic chest pain, which decreases in intensity with leaning forward, and they may also experience febrile episodes. Triphasic pericardial friction rub on the exam is specific and is found during atrial systole, ventricular systole, and diastole. These rubs are commonly auscultated over the left sternal border with the diaphragm of the stethoscope while the patient is leaning forward. An EKG typically reveals diffuse ST elevations with reciprocal depressions in aVR and V1 and PR depressions in V5 to V6 [7,8]. Inflammatory markers such as leukocyte count, erythrocyte sedimentation rate, and C-reactive protein may be elevated as well; however, these may be normal in the hyperacute phase. Viral studies are not routinely completed as the results do not significantly alter the management. Autoimmune and infectious workup is recommended if suspicion is high.

In addition, a transthoracic echocardiogram is recommended for all patients. It is often normal unless a pericardial effusion is present, which supports the diagnosis of pericarditis but is neither sensitive nor specific [4,9]. Cardiac magnetic resonance imaging (MRI) can be pursued if the echocardiogram is nondiagnostic, or if there are concerns about constrictive pericarditis or myopericarditis. On cardiac MRI, the involved inflamed pericardium will appear bright and thick on T2-imaging due to edema and enhancement after gadolinium contrast administration.

If a patient is found to be hemodynamically compromised due to cardiac tamponade, a therapeutic pericardiocentesis is beneficial. However, it is of low yield when used for diagnostic studies. Pericardiocentesis along with pericardial biopsy may be considered in complicated cases refractory to standard treatment for more than three weeks [10-12]. Myopericarditis must be evaluated if a patient is found to have persistently elevated serum troponin levels. Pericarditis is diagnosed when two of the following criteria are met: typical chest pain, pericardial friction rub, characteristic electrocardiogram changes, and new or worsening pericardial effusion [9].

Management is typically colchicine and nonsteroidal anti-inflammatory drugs (NSAIDs). Glucocorticoids are reserved for patients with contraindications to NSAIDs [4]. Colchicine is used for at least three months, and NSAIDs are tapered weekly once symptoms are resolved to prevent recurrence. Gastrointestinal protection with a proton pump inhibitor should be offered to those older than 65 years, history of peptic ulcer disease, or concurrent use of aspirin, anticoagulants, or steroids. Furthermore, strenuous activity should also be restricted until symptom resolution [11]. Inpatient treatment of the patient is recommended if febrile, no acute onset of chest pain, hemodynamic compromise, large pericardial effusion, immunosuppression, use of vitamin K antagonists, acute trauma, and suspicion for myopericarditis. 

## Conclusions

Although cases of acute pericarditis have been reported in Graves’ disease, this case is significant demonstrating the hyperacute phase of pericarditis due to thyrotoxicosis, with no characteristic findings supporting Graves’ disease. It is crucial to detect pericarditis and thyrotoxicosis early and treat promptly to improve patient outcomes. 
